# Brain at Work and in Everyday Life as the Next Frontier: Grand Field Challenges for Neuroergonomics

**DOI:** 10.3389/fnrgo.2020.583733

**Published:** 2020-10-27

**Authors:** Frederic Dehais, Waldemar Karwowski, Hasan Ayaz

**Affiliations:** ^1^ISAE-SUPAERO, Université de Toulouse, Toulouse, France; ^2^School of Biomedical Engineering, Science and Health Systems, Drexel University, Philadelphia, PA, United States; ^3^Computational Neuroergonomics Laboratory, Department of Industrial Engineering and Management Systems, University of Central Florida, Orlando, FL, United States; ^4^Drexel Solutions Institute, Drexel University, Philadelphia, PA, United States; ^5^Department of Psychology, College of Arts and Sciences, Drexel University, Philadelphia, PA, United States; ^6^Department of Family and Community Health, University of Pennsylvania, Philadelphia, PA, United States; ^7^Center for Injury Research and Prevention, Children's Hospital of Philadelphia, Philadelphia, PA, United States

**Keywords:** neuroergonomics, cognition, mobile brain imaging, neurotechnology, human system interaction, neuroscience

## Introduction

The understanding of the brain functioning in the real world is the next frontier: discovering its operational principles, architectural design, and internal mechanisms are attributed as a significant opportunity to advance human civilization (National Academy of Engineering, 2008). How low-level brain processes translate into cognition is one of the greatest unsolved questions. Although science and engineering enabled our understanding of subatomic particles, the formation of solar systems, and the molecular building blocks of nerve cells, it has yet to explain how consciousness and natural intelligence emerge from the electrical and chemical activity of neurons. We need new technologies and novel approaches to study and understand the brain in the wild.

Driven by the national and international grand funding programs such as the BRAIN initiative and Europe's Human Brain Project, it is expected that our understanding of the human brain function and the tools to record and alter brain activity and treat brain diseases will be revolutionized in the upcoming decades (National Institutes of Health, 2014). Existing studies with traditional approaches have accumulated overwhelming knowledge but are limited in scope, i.e., only in artificial lab settings and with simplified tasks. Hence, accurate measurement and precise modulation of the brain activity in a diverse array of everyday tasks is an urgent and needed capability to move neuroengineering and neuroscience to the next level: that is to enable practical clinical and translational research that will form the basis of an entirely new industry of neurotechnologies.

As an interdisciplinary new field, *neuroergonomics* aims to fill this gap: Understanding the brain in the wild, its activity during unrestricted real-world tasks in everyday life contexts, and its relationship to action, behavior, body, and environment. Neuroergonomics is at the intersection of neuroscience, engineering, psychology, philosophy, and human factors. Originating from the use of scientific thinking in the design of tools, technology and of working environments, neuroergonomics represent the next frontier and builds on the research innovation and applications of human performance, cognitive engineering and cognitive neuroscience (Parasuraman, 2003; Karwowski, 2005; Posner, 2012). By utilizing combined approaches, hybrid methods, and domain expertise/knowledgebase, to investigate uncharted scientific territories, neuroergonomics is posed to contribute to each of these fields. Neuroergonomics has the potential to advance our overall understanding of brain with practical implications in diverse sectors such as healthcare, education, transportation, manufacturing, entertainment, communication and everyday life at large (Parasuraman, 2003, 2011; Parasuraman and Rizzo, 2007; Parasuraman et al., 2012; McKendrick et al., 2015; Ayaz and Dehais, 2019).

## New Emerging Field: Neuroergonomics

The efforts to understand the nature of the brain in human performance originated with Hippocrates (c.469–370 BC) (Finger, 2000). Today, neuroscience applies various levels of analysis in investigating human brain activity, including molecular neuroscience, cellular neuroscience, systems neuroscience, behavioral neuroscience, and cognitive neuroscience, with an overall premise that the activity of the brain creates what is known as the human mind (Bear et al., 2020). The understanding that knowledge of human brain functioning is essential and critical for advancing the study and human-systems at work and in everyday life led to the emergence of neuroergonomics as a separate scientific discipline.

Neuroergonomics, as a field of research, has emerged at the very end of the 20th century with the aim to “study the brain at work and in everyday life.” The term was initially proposed by Raja Parasuraman and this new discipline was progressively matured and formalized during the following decade (Hancock and Szalma, 2003; Karwowski et al., 2003; Parasuraman, 2003; Sarter and Sarter, 2003). The birth of this discipline constitutes the achievement of the visionary researchers who sought to innovate by combining theoretical advances in cognitive neuroscience and brain imaging, bio-engineering, genetics, computer science and human factors to better understand human performance in the real world. As reminded by Hancock (2019), neuroergonomics finds its roots in research initiated by researchers concerned with the measure of the neural correlate underlying vigilance, engagement, attention, and multitasking (Parasuraman, 1979; Wickens et al., 1983). Neuroergonomics has grown from the root of the early work on functional neuroimaging (Ogawa et al., 1990; Chance et al., 1993), brain computer interfaces (Vidal, 1973), neurofeedback (Lubar and Shouse, 1976) and neurostimulation (Magnusson and Stevens, 1914; Nitsche and Paulus, 2000). This research paved the way to define a novel framework to go beyond the traditional subjective and behavioral approach sometimes promoted by the human factors and ergonomics community.

Indeed, neuroergonomics proposition is to open the “black box” and to investigate the neurocognitive processes supporting human performance outside the laboratory. This approach to neuroscience offers insight into global neural processing that cannot be achieved using the typical lab settings and artificial tasks. One can propose three main pillars of neuroergonomics: (1) neuroergonomics theory, (2) neuroergonomics abstraction, and (3) neuroergonomics design (Karwowski, 2013). Neuroergonomics theory focuses on the ability to identify, describe, and evaluate human brain signatures and neural markers of human performance, including brain-system interactions in the context of work and technology. Neuroergonomics abstraction refers to the ability to use the neural signatures and relevant brain-system interactions to make predictions about human performance that can be validated in the real world of everyday activities. Finally, neuroergonomics design considers implementing knowledge about the human brain necessary to develop systems that satisfy individual compatibility requirements from the neural processing point of view. Given the above, the neuroergonomics design process can be represented as mapping human brain capabilities and limitations to system-technology-environment requirements and affordances.

**Figure 1:**
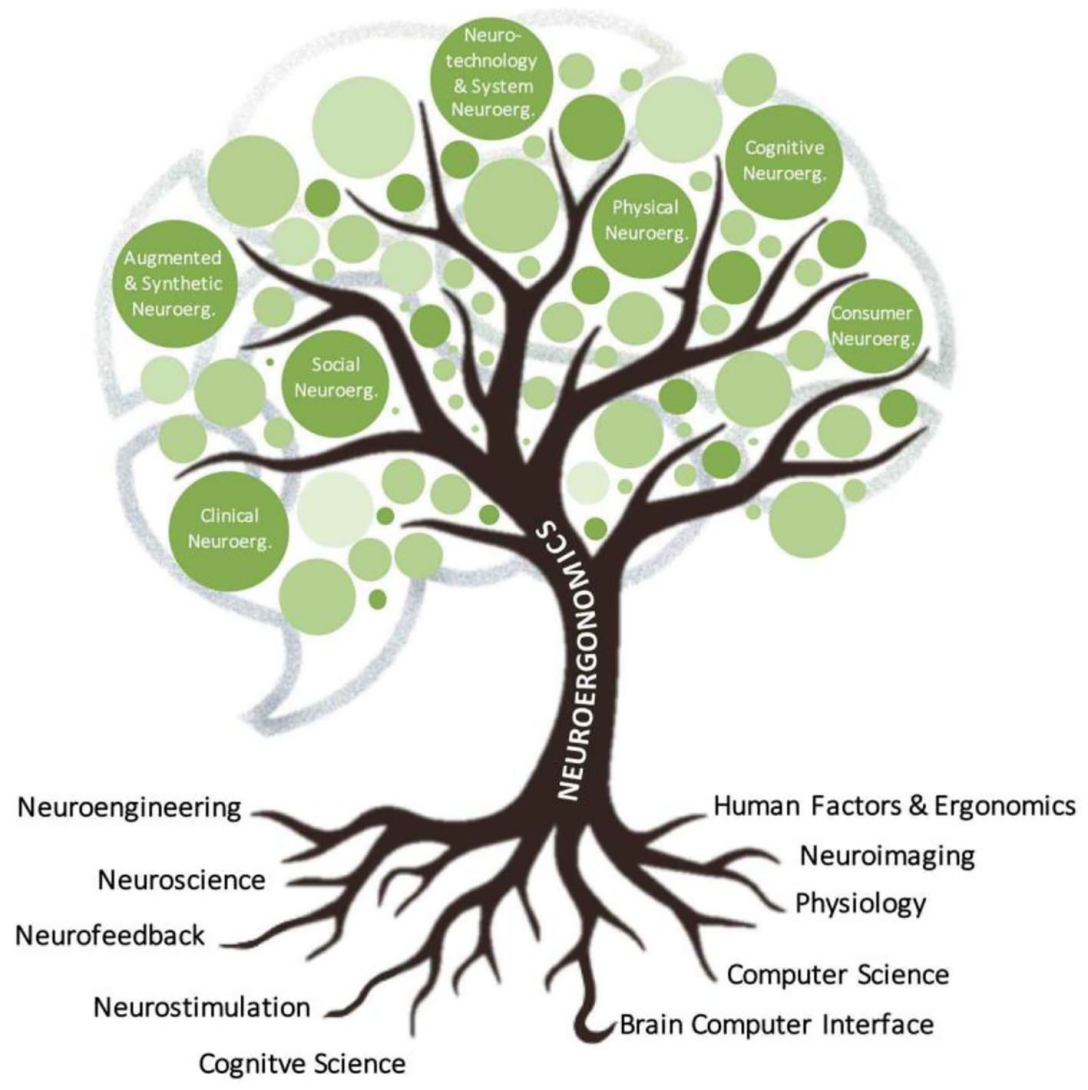
The development and growth of neuroergonomics.

To meet this challenge, neuroergonomics has forged its own mobile cutting-edge tools benefiting from the technological advances in highly portable brain imaging, signal processing, artificial intelligence and increased computational power. Within the last decade, this emerging discipline has demonstrated potential to advances our understanding of brain with practical applications in diverse sectors such as medicine, education/training, aviation, automotive, manufacturing, administration, entertainment, communication and everyday life at large (Parasuraman, 2011; Parasuraman et al., 2012; McKendrick et al., 2015; Gramann et al., 2017; Ayaz and Dehais, 2019). Neuroergonomics has now bloomed and grown to several branches such as cognitive, physical, social, consumer, clinical, augmented & synthetic, and neurotechnology & system neuroergonomics (see Figure 1) to reach new heights in the understanding of our *brain at work and in everyday life situation*.

Here, we identify seven specific sub-domains of neuroergonomics: (i) Cognitive neuroergonomics is concerned with the neural dynamics information underlying perception, attention, memory, emotion, cognitive control and decision during interaction with technical systems at work or in everyday life, using both established neuroimaging approaches and mobile brain imaging methods. (ii) Physical neuroergonomics deals with the human brain in control of muscular performance, movement, and brain-body interactions in conditions of health, workplace, fatigue, training, injury, and disease states. (iii) Social neuroergonomics focuses on how people perform social interaction with other individuals, automation, and autonomy for a diverse spectrum, including teaming, communication, trust, collaboration, competition, or interacting in different ways to share physical or cognitive tasks. (iv) Consumer neuroergonomics research has a strong focus on products, services, and systems on the assessment of their effects on well-being or performance, as well as optimization of design and overall evaluation of any human-interfacing artifact. (v) Augmented & synthetic neuroergonomics focuses on the use of simulations/mixed/virtual reality techniques as well as brain stimulation for cognitive enhancement with or without neuroimaging to investigate and augment brain functioning (e.g., learning/training, perception, affective states) in the real world. (vi) Neurotechnology and Systems neuroergonomics consider all aspects of neurotechnology, which is a topical category of technology where system design incorporates neural principles or directly interfaces with signals from the brain and body. Systems neuroergonomics aims to integrate approaches from neuroergonomics into the design, development, and management of complex systems over their life cycles. And, finally (vii) Clinical neuroergonomics encompasses the utilization of mobile neurotechnologies for brain health and performance across the lifespan as well as prognosis, diagnosis, or personalized treatment of neurological and psychiatric disorders, from hospital to out-patient-clinical settings and particularly toward home settings.

## The Challenges

The exploration of the brain functioning “in the wild” requires mastering knowledge in many technical and theoretical concepts. Neuroergonomics research cannot be achieved through the prism of a single domain. For instance, understanding the brain is not a purely scientific or engineering problem as some may think. The brain needs to be analyzed through its physical, biological, psychological and sociological dimensions. As portable neuroimaging devices become more “plug-and-play,” widespread and cheap, a common misunderstanding may arise that neuroergonomics is just about putting sensors and collecting data on the human brain. The discipline of neuroergonomics should be driven by research questions, theories, concepts, and hypotheses together with well-designed protocols, adequate metrics, and well-thought through statistical procedures and other analytical and quantitative approaches. Progress in this field also requires researchers to work as teams in a truly transdisciplinary spirit, following sound ethical principles. This is how brain research will prove to be efficient, reproducible, and will benefit society at large.

### Challenge 1: Methodological Considerations

The first main challenge for neuroergonomics is to implement an innovative methodology to avoid the pitfalls of reductionist approach leading to place participants facing repetitive and boring artificial tasks causing degradation of data due to attentional and motivational effects as well as investigating task load with realistic/real-world complex cognitive tasks, with studies ranging from pilots, air traffic controllers, surgeons, car drivers, teacher-students in classroom to pedestrians navigating outdoors (Ayaz et al., 2012; Mühl et al., 2014; McKendrick et al., 2016; Arico et al., 2017; Unni et al., 2017; Bevilacqua et al., 2018; Di Flumeri et al., 2018; Gateau et al., 2018; Callan and Dehais, 2019; Djebbara et al., 2019; Modi et al., 2019; Wunderlich and Gramann, 2020). Thus, the challenge for neuroergonomics is to design an engaging ecological paradigm while assuring a high experimental control level. High-quality neuroergonomics research should proceed by conducting a continuum of experiments starting with controlled protocols with high spatial resolution devices (e.g., fMRI, MEG) progressing to more ecological experiments in dynamic micro-worlds using portable devices that are portable but with lower accuracy, to eventually conducting less controlled experiments in a realistic simulated environment and the real world (Ayaz and Dehais, 2019). This implies the use of complex and continuous natural stimuli (e.g., videos, speech, simulator) and the implementation of appropriate signal conditioning and statistical methods to decode the associated brain response on the fly (see, for example, Wong et al., 2018). Much progress has to be made in this direction, including the design of novel protocols and methods to investigate individual differences. This is an important issue as it may affect the sample size, induce biases of the results and the interpretation at the group level, and decrease single-trial classification accuracy. This is also a relevant to the design of personalized products, services, and neuroadaptive technologies and to improve the efficiency of neurostimulation and neurofeedback.

### Challenge 2: Concepts, Measures, and Metrics

Another challenge for neuroergonomics is to develop and shape its own concepts rather than borrowing constructs from other fields. Following this perspective, efforts should be put toward defining new concepts to assess and predict human performance *per se*. For example, most of the research to-date has focused on the measurement of mental workload, but this transactional construct remains difficult to operationalize despite decades of research and more than 200,000 publications since the early 2000s'. As a measure, the mental workload is highly sensitive to inter and intra-individual variability and is limited to providing a non-specific and global indicator rather like a thermometer. However, unlike a thermometer, it does not give access to an absolute and reliable assessment of degraded performance. We advocate rather to identify neurophysiological, physiological and behavioral markers that specifically account for degraded mental states ranging from task disengagement (e.g., mind wandering, effort withdrawal) to task over-engagement (e.g., attentional tunneling, perseveration, inattentional blindness, and deafness see Dehais et al., 2020 for a comprehensive review). This approach will allow us to design neuroadaptive technology to monitor the mental states and trigger appropriate cognitive-countermeasures to mitigate their deleterious effects on human performance.

There is also a myriad of methods to measure cerebral activity, and new tools and innovative approaches are published every year. For instance, a recent and exciting trend is to implement connectivity metrics over fMRI, MEG, EEG, or fNIRS signals to identify neural pathways and brain dynamics (Farahani et al., 2019). It offers exciting prospects for hyperscanning purposes to study social cognition at the cortical level. Researchers have a choice between model-free vs. model-based, Granger-based, phase-based, information-based, or high-order and non-linear methods. Each of these methods can be implemented via different formalisms (e.g., phase slope index, phase-locking factor, phase-locking values). There is a critical need to establish a consensus and recommend that authors benchmark these methods when reporting their results rather than publishing only the successful ones.

### Challenge 3: Improving Portable Sensors

The rapid evolution of personal electronics in the last quarter century has seen remarkable innovation in computers, digitization of sensors and adoption of wearable sensing technologies. Data that could only be collected in laboratory environments is nowadays more accessible, affordable, and easily integrated into popular electronics and other smart devices. This technological development has moved quickly to meet a growing demand for health-analytics, driven by individuals who wanted to learn more about themselves. While, modern activity trackers have introduced physiological measurements such as heart-rate to measure physical exertion or correlates of stress, these measures are fundamentally non-specific, thereby leaving a picture that is far from complete. In order to expand on the features available currently and elevate the role of continuous monitoring solutions in work and at home, the application of wearable sensors must be expanded to new areas and nowhere is there more untapped potential than in the brain (Curtin and Ayaz, 2018).

New applications are now possible due to wearable and mobile nature of portable neuroimaging like investigating neural correlates of spatial navigation and movement (Djebbara et al., 2019), multi-brain interaction (Liu et al., 2017), practical brain-computer interfaces (Zander et al., 2016) and brain to brain interfaces (Jiang et al., 2019). In fact, portable neuroimaging miniaturization efforts started early 2000s (Ayaz et al., 2013; Yücel et al., 2017) and the latest generation of mobile battery operated and wireless systems have allowed monitoring brain activity and investigating cognition-in-action, in increasingly realistic and real-world settings like participants walking outdoors (McKendrick et al., 2016), students in classroom (Poulsen et al., 2017), air-traffic-controllers working on radars (Ayaz et al., 2012), surgeons performing operations (Shewokis et al., 2017; Modi et al., 2019) to pilots flying aircrafts up in the sky (Gateau et al., 2018).

Technical progress has led to the design of portable wireless devices that allow reasonable degrees of freedom of movement to measure brain activity under increasingly naturalistic settings. The development of dry electrodes expanded rapid out-of-lab applications for EEG and has been shown to provide useable signal quality (Zander et al., 2017). Moreover, for optical brain imaging, fiber-less flat sensors were able to increase the surface area of light detectors to increase resistance for motion artifacts, much needed for mobile applications (Ayaz et al., 2013; Wang et al., 2017). However, there are still obstacles plaguing the widespread use of portable and ultra-portable sensors. Several new directions have to be taken to improve and generalize the use of portable neuroimaging for everyday life situations, and specifically at home settings and in clinical applications. For instance, dry-electrode and fiber-based sensors inevitably lead to significant discomfort for participants after an hour. New mobile solutions such as unobtrusive cEEgrid, in-hear electrodes, miniaturized prefrontal adhesive-optodes could allow to overcome these issues. However, one should be aware that these solutions reduce their potential to account for the complexity of the brain. EEG and fNIRS still face some inherent limitations, respectively, in terms of spatial and temporal resolutions. A recent trend is to perform concurrent fNIRS and EEG recording to overcome each other measurement weaknesses and to give a better insight on neurovascular coupling. Again, the combination of these techniques has a cost in terms of setting time, weight, and discomfort for participants. These settings generally result in a non-ideal compromise in terms of signal quality either for fNIRS or for EEG. Hardware should be developed to fully integrate these sensors in a non-intrusive fashion. Futuristic novel approaches are under development, such as holographic optical brain monitoring re-forms light passing through the body may also revolutionize the scanning of the brain in the real world. Similarly, portable magnetoencephalography portable devices are starting to be developed and becoming a new complementary media to investigate human cognition (Boto et al., 2018).

### Challenge 4: Advancing Brain Stimulation

Non-invasive brain stimulation techniques such as transcranial magnetic stimulation (TMS) and variants of transcranial electrical stimulation (tES) such as transcranial direct current stimulation (tDCS) can alter brain function by positioning the actuator devices over the scalp. Such neuromodulation approaches have led to a proliferation of research on the brain and cognitive augmentation, both in healthy adults and in patients with neurological or psychiatric disease. Neuromodulation has shown a lot of promise in the treatment of heterogeneous psychiatric disorders and TMS is already FDA approved for multiple indications: major depression, migraine pain, and obsessive-compulsive disorder. The tES systems have the potential for portability and, new methodological developments (Knotkova et al., 2019) offer new vistas for research and clinical use and even at-home settings (Charvet et al., 2020). Furthermore, integration of these technologies with neuroimaging provides opportunities for read-write brain-computer interfaces, that can both monitor and alter brain activity, (see Cinel et al., 2019 and Rao, 2019 for descriptions and Jiang et al., 2019, for a closed loop multi-brain example) as well as rehabilitation for diverse clinical indications (Teo et al., 2016). There are already best-practices guidelines and systematic reviews for multimodal use: tDCS+fMRI (Esmaeilpour et al., 2020), TMS+fNIRS (Curtin et al., 2019), TMS+EEG (Darmani and Ziemann, 2019).

Moreover, newer techniques have been emerging. Focused ultrasound (FUS) promises to bring together strong features of TMS and tES, high spatial resolution and wearable mobile footprint, respectively, into a single system. So far it has been studied with animal models, and a few human studies have been reported. There are still significant engineering and safety challenges before effective research systems could be available.

A more futuristic neuromodulation technique is optogenetics: a combination of genetic and optical methods for targeted and fast control of neural activity (Deisseroth, 2011). Optogenetics utilizes light for milli-second resolution speed and cell-type specific precise control for neurons. As it requires viral injection to modify the genetic material of target neurons (to insert light-sensitive receptor protein on the cell membrane), this technique cannot be used on humans and is not currently compatible with neuroergonomic approach. However, it has become a workhorse of neuroscience to understand diverse neural systems both in healthy and disease animal models.

Recent trends in neuroergonomics and neural engineering have used neurotechnologies to enhance various human capabilities (cognitive function like attention, decision-making, mood), and treat neurological and psychiatric disorders (Valero-Cabre et al., 2017; Cinel et al., 2019). Neurotechnologies for brain stimulation are evolving rapidly, and new advances in the techniques and applications are expected in near to mid-term. However, research is still needed to assess the effectiveness of such technology as some studies found that effects remain inconsistent (Bestmann et al., 2015; Horvath et al., 2015), and new guidelines for realistic environments are emerging only very recently (Bikson et al., 2020; Charvet et al., 2020).

### Challenge 5: Artificial Intelligence for Neuroergonomics

Another significant development that will have a substantial impact on future advances in neuroergonomics is the rapid progress in artificial intelligence (AI) (Jason, 2017). Artificial intelligence technology, particularly machine learning (ML), has become ubiquitous for solving many complex problems in our daily lives (Bengio et al., 2013). Many AI approaches, including, for example, Support Vector Machine (SVM) and Fisher Linear Discriminant (FLD), have been successfully used for the assessment of brain states based on fMRI data (Mourao-Miranda et al., 2005). The analysis of fMRI scans by means of SVM has also been applied to a sensory-motor task (Wang et al., 2007). Furthermore, it was demonstrated that the functional brain analysis with neurophysiological interpretation could be facilitated by transforming neural networks “backward models” into “forward models” which is applicable for both EEG and fMRI experimental data (Haufe et al., 2014; Li et al., 2018). Deep learning (DL), a subset of ML, has shown remarkable progress in recent years, including applications to assessment of human performance, with a variety of applications such as image classification, speech recognition, or natural language processing (LeCun et al., 2015; Schmidhuber, 2015). Recently, DL with convolutional neural networks has been successfully applied for EEG decoding and visualization purposes (Schirrmeister et al., 2017). The use of such an approach remains challenging because neuroergonomics experiments generally lead to collect small data samples thus limiting the application of DL techniques. We encourage researchers to share their data with the community following the Brain Imaging Data Structure (BIDS) recommendations so as to constitute very large database to improve our understanding of the brain via AI. Such big data of brain could enable larger scale teamwork across interdisciplinary teams and multi-site collaborations.

Recent advances in the theory and applications of explainable artificial intelligence (XAI) to neuroscience (Fellous et al., 2019), is of high relevance to the field of neuroergonomics. Humans beings are already interacting with robots and AI-based algorithms, and this trend will continue to increase. It is of great importance to ensure transparency and explainability of these artificial decisional systems and their evolution over time to improve trust and optimal human-machine teaming. Currently, the main areas of XAI research in the domain of neuroscience include (1) identifying how explainable learning solutions can be applied, (2) developing a community of scholars working with XAI, and (3) stimulating an open exchange of data and theories. In the near future, it will be possible to apply XAI techniques for intelligent decoding and the modulation of behaviorally activated brain circuits to improve our understanding of human behavior in real-world settings. For example, Searchlight, a sophisticated form of XAI approach, has been developed for the visualization of fMRI, allowing to identify differences in the regional spatial activity pattern of the brain under a variety of experimental conditions (Kriegeskorte et al., 2006). Recently, the potential value of XAI in the field of neurostimulation was demonstrated by Fellous et al. (2019). However, as recently discussed by Arrieta et al. (2020), the field of XAI faces many challenges, including setting the objective metrics on what constitutes a good explanation. For example, Páez (2019) noted that the term “explanation,” as it is currently used in XAI, does not share the properties that are attributed to explanations in epistemology and the philosophy of science, and that the interpretative AI models can provide false assurances of comprehensibility. Furthermore, the barrier of explainability brought by sub-symbolism of deep neural networks initiated the discussion of responsible artificial intelligence, i.e. the ways to implement AI methods with fairness, model explainability, and accountability (Leslie, 2019; Arrieta et al., 2020). Finally, Byrne (2019) pointed out that XAI could benefit from including the rich knowledge in cognitive science about human reasoners' cognitive capacities.

This approach can provide promising prospects for the challenges proposed by the National Academy of Engineering (National Academy of Engineering, 2008) to “reverse engineer the human brain.” The realization of such a challenge requires a much better understanding of brain structure and its cognitive functions, and, among other advantages, would lead to the development of general-purpose artificial intelligence, facilitate human learning, improve methods for diagnosing, treating, and monitoring mental illnesses in a personalized manner, or develop a variety of neuroprosthetics (Roysam et al., 2009). It was also observed that the reverse-engineering the brain is critical to understanding the human mind that will have a profound impact on the future advances in technology, health, and society at large. Clearly, neuroergonomics discipline can contribute to and benefit from the realization of this grand challenge.

### Challenge 6: Cognitive Freedom, Privacy, and Ethics in the Age of Neuroergonomics

The advances of neurotechnologies that can record and alter brain activity provide increasingly powerful tools for neuroergonomists and allied professionals and, are rapidly transforming research with implications for everyday life. It is clear that these more powerful methods mean more responsibilities for neuroergonomists. This calls for a new age “neuroergonomics philosophy” as recently defined by Onaral (2021) and informs rethinking of the “neuroergonomic ethos.”

The field of neuroethics emerged in early 2000s triggered with the awareness of advances in cognitive neuroscience (Farah, 2005). Neuroethics encompasses a large and varied set of issues, from practical considerations, like use of neurotechnology or the data, to more philosophical issues. Several ethical and philosophical issues regarding have been recently discussed (Levy, 2007; Giordano and Gordijn, 2010; Farah, 2015; Amadio et al., 2018; Zuk et al., 2018). It can be argued that neuroergonomics faces similar ethical and philosophical challenges as cognitive neuroscience. For example, Farah (2005) and Farah (2015) discussed the implications of the developments in neurotechnology for individuals and society, including the philosophical concerns, including the impact of cognitive neuroscience on the way we think about ourselves as persons, the issue of moral agents, and spiritual beings, the nature of mind, and, ultimately, how, neuroscience will shape the future of individuals and society at large. Recently, Amadio et al. (2018), discussed several neuroethics questions to guide ethical research concerning the potential impact of a model or neuroscientific account of disease on individuals, communities, and society. For example, among many, the following set of considerations, which are also relevant to the field of neuroergonomics, have been posed:

- What are the possible unintended consequences of neuroscience research on social stigma and self-stigma?
- Is it possible that social or cultural bias has been introduced in research design or in the interpretation of scientific results?
- How can human brain data (e.g., images, neural recordings, etc.), and the privacy of participants from whom data is acquired, be protected in case of immediate, or legacy use beyond the experiment?
- What are the requisite or minimum features of engineered neural circuitry required to generate a concern about moral significance?
- Are the ethical standards for research conduct adequate and appropriate for the evolving methodologies and brain models?
- How could brain interventions impact or reduce autonomy?
- What measures can be in place to ensure optimal autonomy and agency for participants/users?
- Who will have responsibility for effects (where responsibility has broad meaning encompassing legal, economic, and social contexts)?
- In which contexts might a neuroscientific technology/innovation be used or deployed?

New ethical challenges that were hypothetical previously are becoming more practical concerns, like who should have access to brain activity patterns, changing personalities with brain stimulation, and mind reading and writing. Considering the growing privacy concerns even about the web surfing behavior, and that social media activity can be used and abused by third parties, the privacy in the age of neuroergonomics is ever vulnerable. Privacy of our brain is the most sensitive data of all as it encapsulates our inner-most thoughts and intents, and the most critical to protect for individuals and society at large. Akin to basic human rights, we should define and protect our neuro rights, fundamental to cognitive freedom and personhood (Ienca and Andorno, 2017).

Responsible neuroergonomics research requires awareness of current neuroethical challenges and continuous interaction among all stakeholders: researchers, ethicists, regulators, lawmakers, lawyers and public at large. As new innovations emerge in neuroergonomic technologies, methods and application, so do new ethical dilemmas. For example, ethical issues specifically arising from the use of portable neuroimaging is recently outlined (Shen et al., 2020), multi-person brain to brain interfacing (Hildt, 2019) and, benefits/risks of neuroenhancement of surgeons (Patel et al., 2020). Recently, Farahany and Ramos (2020) highlighted that neuroethics can foster necessary and beneficial collaborations for responsible neuroscientific discovery. Neuroethics should be an integral part of neuroergonomics, and with the use of foresight, open communication, strategic planning, we can all truly benefit from the advances of neuroergonomics research and development.

## Conclusion

As stated by Parasuraman (2003), the focus of neuroergonomics is on the investigation of the neural bases of mental functions and physical performance in relation to technology, work, leisure, and a broad set of real-world settings, including, e.g., health care, transportation, and many other areas of human endeavor in the wild. As a unique new discipline, neuroergonomics should help to increase our knowledge about the role of the brain in shaping the complex inter-relationships between humans (human capacities and limitations), their every-day life social and natural environment, technology (products, machines, devices, processes), and broadly-defined work systems (business processes and organizational structures).

In our view, neuroergonomics, as a fast-growing field of research, will have robust theoretical and practical implications for discovering useful and impactful knowledge about human neural capacities and limitations in the context of our everyday activities at work, home, and at leisure. We look forward to facilitating the development of this new field of study for the ultimate benefit of improving the quality of life for the billions of people around the world, and we hope that you will join us on this exciting journey.

## Author's Note

This is a Field Grand Challenge submitted by the Field Chief Editors.

## Author Contributions

All authors listed have made a substantial, direct and intellectual contribution to the work, and approved it for publication.

## Conflict of Interest

The authors declare that the research was conducted in the absence of any commercial or financial relationships that could be construed as a potential conflict of interest.
